# Dietary Patterns in Secondary Prevention of Heart Failure: A Systematic Review

**DOI:** 10.3390/nu10070828

**Published:** 2018-06-26

**Authors:** Gabriela dos Reis Padilha, Karina Sanches Machado d’Almeida, Stefanny Ronchi Spillere, Gabriela Corrêa Souza

**Affiliations:** 1 Nutrition Graduate Course, Universidade Federal do Rio Grande do Sul, UFRGS, Porto Alegre 90035-003, Brazil; gabrielarpadilha@outlook.com; 2 Heart Failure and Transplant Group—Hospital de Clínicas de Porto Alegre, Porto Alegre 90035-003, Brazil; karinasmdalmeida@gmail.com (K.S.M.d.); spillerestefanny@gmail.com (S.R.S.); 3 Nutrition Graduate Course, Universidade Federal do Pampa, UNIPAMPA, Itaqui 97650-000, Brazil; 4 Multiprofessional Health Residency Program/Hospital de Clínicas de Porto Alegre—HCPA, Porto Alegre 90035-003, Brazil; 5 Post-Graduation Program in Food, Nutrition and Health, School of Medicine, Universidade Federal do Rio Grande do Sul, Porto Alegre 90035-003, Brazil

**Keywords:** heart failure, dietary patterns, Mediterranean diet, DASH

## Abstract

Background: Diet is an important factor in secondary prevention of heart failure (HF) but there is still no consensus as to which dietary model should be adopted by this population. This systematic review aims to clarify the relationship between dietary patterns and secondary prevention in HF. Methods: We searched the Medline, Embase and Cochrane databases for studies with different dietary patterns and outcomes of secondary prevention in HF. No limitation was used in the search. Results: 1119 articles were identified, 12 met the inclusion criteria. Studies with Dietary Approaches to Stop Hypertension (DASH), Mediterranean, Hyperproteic and Low-carb diets were found. The DASH pattern showed improvement in cardiac function, functional capacity, blood pressure, oxidative stress and mortality. The Mediterranean diet had a correlation with inflammation, quality of life and cardiac function but just on cross-sectional studies. Regarding the Hyperproteic and Low-carb diets only one study was found with each pattern and both were able to improve functional capacity in patients with HF. Conclusions: DASH pattern may have benefits in the secondary prevention of HF. The Mediterranean diet demonstrated positive correlation with factors of secondary prevention of HF but need more RCTs and cohort studies to confirm these effects. In addition, the Hyperproteic and Low-carb diets, despite the lack of studies, also demonstrated positive effects on the functional capacity in patients with HF.

## 1. Introduction

Heart Failure (HF) is a systemic syndrome characterized by reduced cardiac output and/or high intracardiac pressure, compromising the heart’s ability to maintain adequate oxygenation of tissues [1,2]. It is considered the final pathway of heart diseases, causing a reduction in longevity and decline in patients’ quality of life, besides being associated with disorders of muscle structure and metabolism, which may increase intolerance to exercise or physical effort, fragility and mortality [3].

The prevalence of HF is around 1–2% in the adult population in developed countries and increases by more than 10% in individuals over 70 years old. Moreover, the risk of developing HF at age 55 is around 33% for men and 28% for women [2]. There are several etiologies associated with HF, including coronary artery disease (CAD), dilated cardiomyopathy, hormonal diseases, infections (such as Chagas’ disease) and drug use [2]. Among the risk factors, we can highlight: CAD, male gender, physical inactivity, overweight, hypertension, diabetes mellitus (DM), smoking and others [4,5,6].

Secondary prevention in HF consists of medical and pharmacological care and non-pharmacological management [7]. Factors related to the therapeutic education of the patient, including lifestyle modification with physical activity, food intake and weight control, are non-pharmacological strategies used, especially for blood pressure (BP) control, because high levels of BP contribute to the progression of the disease, structural abnormalities and worse prognosis [8,9,10,11].

The literature indicates that adherence to different dietary patterns has an influence on the primary prevention of HF [12], furthermore, nutritionally unbalanced diets can contribute to disease progression [13]. Therefore, this systematic review intends to investigate the risks and benefits induced by feed in the secondary prevention of HF.

## 2. Materials and Methods

This systematic review was conducted according to The Cochrane Collaboration [14] and the Preferred Reporting Items for Systematic Reviews and Meta-Analyzes (PRISMA-P Statement) [15]. This study is registered in the International Prospective Register of Ongoing Systematic Reviews and Meta-Analyzes (PROSPERO) under number CRD42017071223.

### 2.1. Search Strategy and Study Selection

The studies were identified through electronic database searches in Medline (accessed by Pubmed), Embase and Cochrane Central up to December 2017. No filter or limitation was used during the search. References of the selected studies were also analyzed to find other related publications.

Key words and synonyms were used for intervention such as “dietary pattern,” “diet mediterranean,” “dash diet,” “diet vegetarian,” “diet paleolithic,” “diet fat-restricted,” “diet, carbohydrate-restricted”; and keywords and synonymous to the patient as “heart-failure.”

### 2.2. Eligibility Criteria

To assess the relationship between different dietary patterns and secondary prevention of HF, studies with adult individuals with HF were included. The studies should include data comparing groups of patients who had adherence to dietary patterns (DASH, Mediterranean, Low-carb, Hyperproteic, Low-fat, Paleolithic or Vegetarian) and secondary prevention outcomes in HF. The select outcomes were: quality of life, functional capacity, BP, cardiac function, oxidative stress, inflammation and mortality. There was no restriction regarding study design. Experimental studies, those that include children, those who did not present dietary patterns, those who did not present secondary prevention outcomes in HF and those with individuals without HF diagnosis were excluded.

### 2.3. Data Extraction

Titles and abstracts were evaluated by two independently reviewers (G.R.P. and K.S.M.D.). The Kappa index was calculated to assess the agreement between the two reviewers and any discrepancy was resolved by consensus or by a third reviewer (G.C.S.). The reviewers were not blind to author, institutions, or manuscripts journals. Articles that did not provide sufficient information from the title and abstract were included for further evaluation and the reading was done in full. Data extraction and analysis were performed by the same two reviewers. For each study, information on publication data, population characteristics, intervention and comparison group, results and limitations was extracted. The authors of the abstract were contacted by e-mail to provide more information about their research.

### 2.4. Assessment of Bias Risk and Study Quality

Methodological quality was explored using an approach similar to that recommended by Cochrane Collaboration in risk assessment [14]. Quality assessment and risk bias included specific questions for randomized clinical trials (RCTs) and observational studies. The following dimensions were considered: study design, sequence generation, concealment of allocation, blinding, losses and exclusions and description of confounding factors. Risk judgment was assessed using pre-specified study criteria and expressed as “low risk of bias,” “high risk of bias” or “unclear risk of bias” (Table A1).

## 3. Results

### 3.1. Descriptions of Studies

A total of 1119 studies were found and after removal of the duplicates, 1050 were screened by title and abstract. After analysis, only 12 studies were included in the systematic review (Table A2). The agreement between the reviewers was total: Kappa = 1.0.

**Figure 1:**
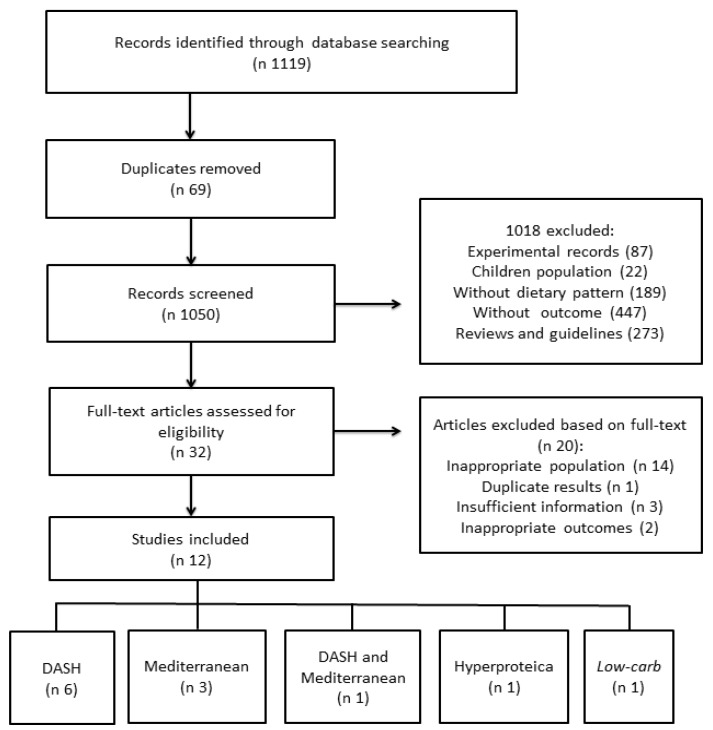
Flowchart for selection of articles.

Four RCTs, five cohort studies and three cross-sectional studies were used for the systematic review (Figure 1).

A total of 4.201 participants were included in this review. Of the 12 included studies, six belonged to the gray literature, with only the abstract available [16,17,18,19,20,21]. Of the abstracts, some did not provide data regarding age [16,20], gender [16,19,20,21] and method of evaluating diet adherence [16,21] and none presented the mean of ejection fraction. Among the full-text articles, only one did not have ejection fraction data [22].

Five cohort studies [20,21,22,23,24] and two RCTs [17,25] investigated the effects of adherence to the DASH dietary pattern, being conducted in the United States [17,20,21,23,24,25] and Europe [22]. Regarding the Mediterranean diet, three cross-sectional studies [18,19,26] and one cohort study [22] were included, three of which were from Europe [18,19,26] and one from United States [22]. For the low-carb dietary pattern we included a RCT conducted in Mexico [16]. Finally, for the Hyperproteic diet we found a RCT from the United States [27].

Most studies include patients with chronic HF. One study evaluated ambulatory patients with HF and DM2 (not on insulin therapy) who were overweight (BMI ≥ 27) and were not eligible for transplantation [27]. Four studies evaluated hypertensive patients with HF and preserved ejection fraction [20,21,23,24]. Two studies evaluated patients with systolic HF [19,26]. And one study evaluated postmenopausal women with HF [22]. There were no studies that evaluated the adherence to Paleolithic, Low Fat and Vegetarian diets in the secondary prevention of HF. The follow-up period varied between 21 days and 4.6 years. Table A2 shows more details about the studies included in the systematic review.

### 3.2. Quality and Publication Bias Assessment

Regarding the RCTs, one was considered of high risk for the blindness of the investigator, participants and evaluators [25]. None of the RCTs presented a clear description of concealment of allocation and losses and exclusions. Only one of the cohort studies presented the description of the confounding factors in the adjusted analyzes and the balance between the groups at the start of the study [22]. Moreover, in relation of the cross-sectional studies, one presented a clear description of the confounding factors in the adjusted analysis and the balance between the groups at the start of the study [26] and the other two did not present a clear description of the baseline characteristics of the participants (Table A1).

### 3.3. DASH

Most studies have evaluated the effects of adherence to a DASH diet, totaling seven articles. Two RCTs [17,25], five cohort studies [20,21,22,23,24], one of them evaluated DASH and Mediterranean diets [22] and three of them were published by the same group of researchers [20,23,24].

In the study proposed by Rifai et al. [25], a RCT that included 48 subjects (29 were women) with chronic HF, stage C and functional classes (NYHA) I-III, patients were randomized to follow a DASH diet (n 24) or general guidelines for management of HF (n 24) and all were followed up for 3 months. The mean age was 60 ± 11 years in the intervention group and 64 ± 12 years in the control group. The objective was to evaluate endothelial function, functional capacity and quality of life. A shopping list was provided to the intervention group and weekly food records were applied. In addition to nutritional counseling at the beginning of the study, monthly visits and weekly phone calls were made for orientation. To assess the adequacy of the diet the Folsom index was used [28], which evaluates specific components of the DASH dietary pattern, applied monthly. Endothelial function, measured by large and small arterial elasticity, was better in the intervention group but did not reach statistical significance (*p* > 0.05). Regarding functional capacity, evaluated through the 6-min walk test (measured in meters), no statistically significant difference was found between groups at baseline. However, at the end of the study, the intervention group had a better performance (intervention: 292 ± 124, control: 197 ± 81, *p* = 0.018). The intervention group also obtained a better quality of life index (MLHFQ) after 3 months, when compared to the control group (intervention: 21 ± 15, control: 39 ± 22, *p* = 0.006).

In the RCT conducted by Silver [17], where only the abstract was available, 40 subjects with chronic HF, 18 women, with 40 to 84 years of age, were randomized to follow a DASH diet or usual diet for one month. To evaluate diet adherence, the DASH diet index was used. Arterial compliance and functional capacity were evaluated. At the end of the intervention, it was observed that the group that followed the DASH dietary pattern improved arterial compliance (>4 units Δ, *p* < 0.05) and functional capacity, measured by the 6-min walk test (baseline: 255, after 1 month: 292 m, *p* < 0.05).

The cohort study by Vittos et al. [21] had only the abstract available. This study followed 17 hypertensive individuals with compensated Heart Failure with preserved Ejection Fraction (HFpEF) and 10 healthy controls, for 21 days. The authors did not provide information on the characteristics of the patients, such as gender and age. All subjects received the intervention (DASH diet) and BP and oxidative stress were evaluated. At the end of the study, the HFpEF group presented a reduction in systolic BP (baseline: 158, after 21 days: 140 mmHg, *p* < 0.05) and diastolic BP (baseline: 81, after 21 days: 75 mmHg, *p* < 0.05). Oxidative stress, measured by activity of myeloperoxidase (MPO), decreased in the HFpEF group but remained at levels significantly higher than those found in the control group (baseline: HFpEF = 303.04 ± 18.7, Control = 248 ± 21.3, after 21 days: HFpEF = 282.1 ± 14.9 IU/L, *p* < 0.05).

In the three studies of the same research group, who probably evaluated the same population; hypertensive outpatients with HFpEF and mean age of 72 ± 10 years received a DASH diet for 21 days of follow-up. The sample was composed of 13 individuals (12 were women) in two of these three studies [23,24] and 12 individuals in one of the articles without the description of gender [20]. All foods and beverages were provided to follow the DASH dietary pattern. The adherence was assessed with a 3-day food registry and 24-h urinary sodium and potassium excretion.

The study by Hummel et al. [23] evaluated functional capacity, BP and oxidative stress. After 21 days of follow-up, the functional capacity improved, with a significant increase in the distance walked in the 6-min walk test (baseline: 313 ± 86, after 21 days: 337 ± 91 m, *p* = 0.006). In relation to BP, there was a significant reduction in clinical systolic (baseline: 155 ± 29, after 21 days: 138 ± 22 mmHg, *p* = 0.02) and diastolic BP (baseline: 79 ± 15, after 21 days: 72 ± 8 mmHg, *p* = 0.04) and 24-h ambulatory systolic (baseline: 130 ± 4, after 21 days: 123 ± 4 mmHg, *p* = 0.02) and diastolic BP (baseline: 67 ± 3, after 21 days: 62 ± 3 mmHg, *p* = 0.02). Oxidative stress was evaluated by urinary F2-isoprostanes and the results showed a 31% reduction in F2-isoprostane levels (baseline: 209, after 21 days: 144 pmol/mmolCr, *p* = 0.02) and this reduction closely correlated with urinary sodium excretion (R = −0.19, *p* = 0.57).

Hummel et al., proposed another study [24] to evaluate cardiac function, arterial elastance, ventricular-atrial coupling, viscoelastic/relaxation flexibility and chamber stiffness. After the intervention, the arterial elastance decreased (baseline: 2.0 ± 0.4, after 21 days: 1.7 ± 0.4 mmHg/mL, *p* = 0.007), the improved ventricle-atrial coupling (baseline: 1.5 ± 0.3, after 21 days: 1.7 ± 0.4, *p* = 0.04) and reduced both viscoelastic/relaxation flexibility (baseline: 24.3 ± 5.3, after 21 days: 22.7 beats/min (*p* = 0.03) and chamber stiffness (baseline: 252 ± 115, after 21 days: 170 ± 37 s^−1^, *p* = 0.03). The results indicate that the DASH diet improved left ventricular diastolic function, arterial elastance and ventriculo-atrial coupling, demonstrating an improvement in blood circulation.

The cohort study published in 2014 by Hummel et al. [20], only the abstract was available and the outcome of interest was oxidative stress, evaluated by measurement of endogenous cardiotonic steroids (F2-isoprostanes:Cr, Aldosterone:Cr, marinobufagenin (MBG):Cr, Sodium:Cr (Na:Cr)). The results indicated reduction of oxidative stress: the F2-isoprostane:Cr ratio reduced (baseline: 6.0 ± 2.5, after 21 days: 4.3 ± 1.4 pg/mg, *p* = 0.04), Aldosterone:Cr increased (baseline: 6.7 ± 2.6, after 21 days: 14.2 ± 7.3 pg/mg, *p* = 0.009), the MBG:Cr did not differ significantly from the values at baseline (*p* = 0.91) but the changes (Δ) presented in this parameter correlated closely with the changes in the Na:Cr ratio. By linear regression the ΔMBG:Cr was associated with ΔNa:Cr (β = 23.8, *p* <0.001) and ΔF2-isoprostans:Cr (β = 1.1, *p* = 0.06) independent of ΔAldosterone:Cr.

Levitan et al. [22] developed a cohort that followed 3.215 postmenopausal women with HF enrolled in The Women’s Health Initiative for a median of 4.6 years and observed the general mortality rate associated with adherence to Mediterranean and DASH diets, being the only study that evaluated this outcome. The women who had lower adherence to the DASH diet were classified in quartile Q1 and had a 10% mortality rate. The women with higher adherence were classified in quartile Q4 and had 8.8% mortality. After adjusting for possible confounding factors, higher rates of adherence (Q4) when compared to the lower quartile of adhesion (Q1), were associated with a 16% lower mortality in women with HF (HR = 0.84, 95% CI 0.70–1.00, *p* = 0.01).

### 3.4. Mediterranean

Three cross-sectional studies with Mediterranean diet were found [18,19,26] besides the cohort study of Levitan et al. [22], which evaluated both Mediterranean and DASH diets. The same author conducted all cross-sectional studies, two were abstracts and one complete manuscript. The three studies used the same methodology to estimate food intake and adequacy with the Mediterranean dietary pattern. Food intake was assessed by a food frequency questionnaire (FFQ) and the adequacy to the Mediterranean diet was evaluated using the Mediterranean Diet Score (MDS) [29] with a score of 0–9.

The study conducted by Chrysohoou et al. [18] included 218 patients with chronic HF, 34 were women. The outcome of interest was cardiac function, assessed by echocardiography. The study found a positive association between higher adherence scores to a Mediterranean diet and systolic function (Smv wave: 0.62 ± 0.08, *p* = 0.001, Stv wave: 0.25 ± 0.09, *p* = 0.07) (0.8 ± 0.20, *p* = 0.03), left ventricular ejection fraction (0.58 ± 0, 30, *p* = 0.05) and left atrial ejection fraction (2.20 ± 0.67, *p* = 0.001).

Other study by Chrysohoou et al. [19] included 106 individuals with systolic HF. Outcomes analyzed were circulating inflammatory cytokines (IL-6 and TNF-a) and quality of life evaluated by the Euro-Heart Survey questionnaire. The highest adherence to the Mediterranean diet was inversely related to the circulating levels of inflammatory cytokines (IL-6: R = −0.56 ± 0.168, *p* = 0.004, TNF-a: −0.599 ± 0.281, *p* = 0.047) and quality of life (R = −0.52 ± 0.25, *p* = 0.040) in these patients.

A third study, also of Chrysohoou et al. [26], 372 individuals (58 women), with systolic HF and ejection fraction <40% were included. Systolic and diastolic function of both ventricles were evaluated. Before the adjusted analysis for the possible confounding factors, the Mediterranean diet showed a positive correlation with systolic function (log Smv: R = 0.154, *p* = 0.009) and left atrial ejection fraction (R = 0.133, *p* = 0.041) and negative correlation with diastolic function (log E/AR = −0.24, *p* = 0.001, log Emv/Amv: R = −0.133, *p* = 0.041). However, after adjusting for possible confounding factors, only the log E/A ratio was inversely associated with MDS (*p* = 0.04). The other echocardiographic findings were not statistically significant association with MDS.

In the cohort study already mentioned [22], the mortality rate associated with adherence to the Mediterranean and DASH diets was analyzed. After adjusting for possible confounding factors, women in the quartile of greater adherence to the Mediterranean diet did not show a significant difference in mortality when compared to women with lower adherence (HR = 0.85 CI 95% 0.70–1.02, *p* = 0.08).

### 3.5. Low-Carb

We found only an abstract relating Low-carb diet and secondary prevention in HF [16]. The study design was a RCT, where 123 subjects on outpatient follow-up with stable chronic HF were designated to 4 groups and followed up for 2 months. Group A was an intervention with a low-carb diet and exercise, group B was only low-carb diet, group C was a usual diet with exercise and group D was just usual diet. The analyzed variables were measured at the beginning of the study and monthly at follow-up. The outcomes listed to enter this review were: BP and functional capacity, evaluated by the 6-min walk test.

After 2 months of follow-up, groups A and C showed a decrease in diastolic BP levels (group A: 71.5 ± 2.98 vs. 62.5 ± 1.70 mmHg, *p* = 0.03), group C: 71.76 ± 2.31 vs. 64.69 ± 1.9 mmHg, *p* = 0.04). Also at 2 months, groups A and B improved functional capacity, on the 6-min walk test (group A: 270.37 ± 11.39 vs. 301.78 ± 7.02 m, *p* = 0.08), (group B: 345.77 ± 22.32 vs. 370.07 ± 26.15 m, *p* = 0.05).

### 3.6. Hyperproteic

Of the articles selected for the review only one evaluated the relationship between Hyperproteic diet and secondary prevention in HF [27]. It was a RCT with 14 patients on outpatient follow-up with HF and DM2 (not treated with insulin) with excess weight (BMI ≥ 27) and without eligibility for transplantation. Most patients had a non-ischemic HF (57.1%), with a mean ejection fraction of 26 ± 7.3% and were followed up for 12 weeks. The individuals were designated for 3 groups, one group with Hyperproteic and Hypocaloric (HP) diet: 40% total energy of the carbohydrate, 30% of protein and 30% of lipid; a group with Normoproteic and Hypocaloric (NP) diet: 55% total energy of the carbohydrate, 15% of protein and 30% of lipid; and a group with Usual Diet (UD), without calorie restriction. Visits were made by a nutritionist, where guidelines were given and food list and indicated portions were provided. Adherence to dietary pattern was assessed by means of a 3-day food registry. The outcomes of interest analyzed were: functional capacity and quality of life.

Adhesion for the HP group was classified as “good” (19–22 g protein per day) for one of the 5 individuals; “very good” (23–26 g protein per day) for two and “excellent” (≥27 g protein per day) for other two subjects. At the end of the follow-up, there was an improvement in functional capacity. The HP group increased the distance in the 6-min walk test (HP: 87.5 ± 21; NP: −3.7 ± 21, UD: −42.2 ± 23.5 m, *p* = 0.01) and improved the maximum VO_2_ (HP: 3.1 ± 1.0, NP: −0.3 ± 1.0, UD: −0.3 ± 1.1 mL/kg/min; *p* = 0.003). Regarding quality of life, there was no significant change in general and emotional indexes, however, there was a significant improvement in the HP group for the physical quality of life index (HP: −5.4 ± 7, 0, NP: 2.4 ± 8.9, UD: 5.7 ± 2.5, *p* = 0.022) [27].

## 4. Discussion

The results of our systematic review suggest that DASH diet may have benefits in secondary prevention of HF. The Mediterranean diet had a positive correlation with factors of secondary prevention but just on cross-sectional studies. Furthermore, the Hyperproteic and Low-carb diets, despite the lack of studies, also demonstrated positive effects in patients with HF.

It is well known that DASH diet has an effect on the prevention of cardiovascular diseases [30,31,32,33] and reduction in BP in healthy individuals [31,34] and has been shown to reduce BP also in patients with HF [21,23]. This effect seems to be related to the high levels of potassium, magnesium, calcium and inorganic nitrate of the diet beyond the low levels of sodium [35,36,37].

Regarding decrease in the levels of oxidative stress induced by the DASH diet in patients with HF [20,21,23], an increase in the antioxidant capacity promoted by diet in obese individuals [38] and in obese women with polycystic ovary syndrome [39] was observed (intervention: +98.6 mmol/L, control: −174.8 mmol/L, *p* < 0.001). This effect can be attributed to a large amount of foods rich in antioxidants, such as fruits and vegetables [38] and the high content of magnesium and vitamin C of the diet, since these nutrients have already been shown to increase total antioxidant capacity in healthy young adults [40]. In relation to the changes promoted in cardiac function [17,23,24], the authors suggest that antioxidant compounds may have induce a protective effect on preservation of endothelial function [25]. These findings are in agreement with studies performed in hypertensive adult individuals free of HF, where the reduction of dietary sodium promoted an improvement in the endothelial function and arterial stiffness [41,42].

The 6-min walk test is considered a good measure of functional capacity, physical effort tolerance and prognostic factor in individuals with HF [43]. The studies evaluated in this review [17,23,25] have demonstrated that the DASH dietary pattern may promote improved performance in the test. In addition, there are data in the literature correlating greater functional capacity with lower mortality in this population [44], however, in the included studies, such correlation could not be observed due to the short follow-up.

Adherence to diet can be influenced by factors such as economic and geographic access to food. A study of more than 9000 adults UK residents, analyzed the economic and geographic accessibility to foods that compose the DASH diet and showed that the lower accessibility, mainly economic, was associated with lower adherence to these dietary pattern [45]. The included studies, conducted by the same research group [20,22,23], provided all diet foods to the participants, which could generate an adherence-related bias when we take these factors into consideration. In addition, most of the included studies that assessed the DASH dietary pattern, did not provide detailed data from the control group or had no comparator. The DASH diet is healthy, so we hypothesized that it can overcome the usual patterns of unhealthy diets in various populations, demonstrating the expected benefits of changes in eating habits.

The cohort of Levitan et al. [22] evaluated the intake of patients who adhered to the DASH and Mediterranean diets and showed that a greater adherence to diets was associated to higher intakes of fruits and vegetables, nuts, vegetables, whole grains and fish and reduction of beverage intake sweetened and processed red meat. The authors report that the main limitation of the study was the difficulty in evaluating the intake of sodium, olive oil and liquids. It was observed that individuals with a higher adherence to the DASH diet had a 16% reduction in all-cause mortality [22]. A similar result was observed in a recently published cohort study that followed individuals free of cardiovascular disease at the beginning of follow-up and found that every increment of a standard deviation in adherence to the DASH diet was associated with a 17% reduction in the risk of all-cause mortality after adjusting for confounding variables [46].

Regarding the Mediterranean diet, the same cohort showed a 15% reduction in the mortality rate of individuals with greater adherence, although this value was not statistically significant [22]. These findings differ from those found in the literature available to other populations, which demonstrate a significant correlation between Mediterranean diet and reduction of general and cardiovascular mortality [47,48,49]. A cohort study conducted with more than 4000 elderly Italians found that individuals with greater adherence to a Mediterranean diet had 34% lower all-cause mortality when compared to those with low dietary adherence (RR = 0.66, 95% CI: 0.49–0.90, *p* = 0.0144) [50]. In another prospective cohort performed with a population of West Asia aged 35 years or older that included a similar number of participants, this diet was also identified as a protective factor, since those who were in the highest quartile of adherence were 58% less likely to cardiovascular mortality (RR: 0.42, CI 95%: 0.19–0.96, *p* = 0.02) [51].

The improvement in quality of life associated with adherence to a Mediterranean diet in individuals with HF [19] is in agreement with the literature. This relationship was previously observed in healthy patients by a cohort performed in North America [52] and in another cohort that included more than 11.000 participants in Spain, which identify a significant association between adherence to the Mediterranean diet and improvement of physical and mental health, vitality (β = 0.50, 95% CI: 0.32–0.68) and general health (β = 0.45, 95% CI: 0.26–0.62) [53]. In a study conducted by Bonaccio et al. [54], where this association with quality of life was also observed, the authors attributed this improvement to the total number of antioxidants and fibers present on this dietary pattern.

HF is a disease that impairs cardiac functioning and, therefore, blood supply to the tissues [55], so interventions that improve cardiac contractile function are treatment options. The Mediterranean diet has been associated with improvement in ventricular function in healthy [56] and HF subjects, as may be seen in the study conducted by Crysohoou et al. [18], included in this review. Olive oil, the main component of the Mediterranean diet, had its consumption associated with improved endothelial function in young hypertensive women in an RCT published in 2012 [57]. In addition, olive oil is rich in monounsaturated fatty acids (MUFAs) and phenolic compounds. Phenolic compounds have anti-inflammatory properties [58], which may explain the observed association between diet and lower levels of inflammatory cytokines [19]. But we must consider that these studies, included in this review, were of a cross-sectional methodology, without monitoring the effects of the Mediterranean diet in the long term or comparison with other interventions or controls.

The study by Gonzales et al. [16], who evaluated the effects of a restricted diet on carbohydrates associated with physical exercise in patients with HF, points out physical exercise as the main factor for BP reduction, regardless of diet and the Low-carb diet was shown effective in improving functional capacity, regardless of physical of exercise [16]. Evidence on this dietary pattern in patients with HF is still scarce. The literature search shows that the main outcome investigated is dietary weight loss in different populations. Most of the findings refute the hypothesis of weight loss attributed to the Low-carb diet, indicating that adherence to isoenergetic or hypocaloric diets would have a similar effect, independent of the macronutrient distribution [59,60,61,62,63]. However, review studies demonstrated that following a low-carbohydrate diet may reduce cardiovascular risk factors such as fasting glycaemia, HbA1c, LDL-c, triglycerides and total cholesterol and promote increased HDL-c in individuals with no history of cardiovascular disease but does not promote a significant change in BP [64,65].

Adherence to the Hyperproteic diet, although there are few findings in the literature, promoted an improvement in functional capacity. Subjects in the Hyperproteic diet group increased the distance walked on the 6-min walk test and improved maximal VO_2_ in the study included in this review [27]. According to the authors, this change can be attributed to the loss of adiposity and the hemodynamic improvement of the participants. Furthermore, data from the literature indicate that maximal VO_2_ may be a good prognostic factor independent of the risk of death in HF [66].

### Limitations

After analyzing the studies included in this review, we acknowledge that there are limitations to the interpretation of the data. Many articles had a small sample size and short follow-up time. Divergences and concealment of data from assessment of adherence to dietary patterns were observed. Most studies presented uncertain risk of bias (Table A1) and one of the included RCTs present a high risk in the methodological quality. Moreover, the inclusion of gray literature is a limitation, although it has been an attempt to include all existing data regarding this issue. And we emphasize that there are three studies of the same research group, who probably evaluated the same population. The interpretation of the relationship between Low-carb and Hyperproteic diets in the secondary prevention of HF has as main limitation the lack of studies, since we found only one study for each of the diets.

Authors should discuss the results and how they can be interpreted in perspective of previous studies and of the working hypotheses. The findings and their implications should be discussed in the broadest context possible. Future research directions may also be highlighted.

## 5. Conclusions

In this review, we present the effect of four different dietary patterns on secondary prevention in HF. DASH diet demonstrated to contribute positively to secondary prevention in HF, mainly in relation to cardiac function, functional capacity, oxidative stress, BP and mortality. The Mediterranean diet had a correlation with inflammation, quality of life and cardiac function but just on cross-sectional studies. Hyperproteic and Low-carb diets demonstrate benefits over functional capacity. The studies included in this review can be a starting point to better investigate the relationship between dietary patterns and secondary prevention of HF.

## Appendix A

**Table A1:** Quality assessment of studies.

Study	SD	RSG	AC	Blinding of Research	Blinding of PP	Blinding of OA	LE	Do They Describe Confounders in an Adjusted Analysis?	Do You Assess the Balance between the Groups at the Start of the Study?
Rifai et al. [25]	RCT	Low risk	Unclear risk	High risk	High risk	High risk	Unclear risk	NA	NA
Silver * [17]	RCT	Unclear risk	Unclear risk	Unclear risk	Unclear risk	Unclear risk	Unclear risk	NA	NA
Vittos et al. * [21]	Cohort	NA	NA	NA	NA	NA	NA	Unclear risk	Low risk
Hummel et al., (2012) [23]	Cohort	NA	NA	NA	NA	NA	NA	Unclear risk	Low risk
Hummel et al., (2013) [24]	Cohort	NA	NA	NA	NA	NA	NA	Unclear risk	Low risk
Hummel et al., (2014) * [20]	Cohort	NA	NA	NA	NA	NA	NA	Low risk	Unclear risk
Chrysohoou et al., (2009a) * [18]	Cross-sectional study	NA	NA	NA	NA	NA	NA	Low risk	Unclear risk
Chrysohoou et al. (2009b) * [19]	Cross-sectional study	NA	NA	NA	NA	NA	NA	Low risk	Unclear risk
Chrysohoou et al., (2012) [26]	Cross-sectional study	NA	NA	NA	NA	NA	NA	Low risk	Low risk
Levitan et al. [22]	Cohort	NA	NA	NA	NA	NA	NA	Low risk	Low risk
Gonzales et al. * [16]	RCT	Unclear risk	Unclear risk	Unclear risk	Unclear risk	Unclear risk	Unclear risk	NA	NA
Evangelista et al. [27]	RCT	Unclear risk	Unclear risk	Unclear risk	Unclear risk	Unclear risk	Unclear risk	NA	NA

* Abstract; NA: not applicable; SD: study design; RSG: random sequence generation; AC: allocation concealment; PP: participants and personnel; OA: outcome assessment; LE: losses and exclusions.

**Table A2:** Characteristics of studies.

Study	SD	Population (N)	Age (years)	Intervention	Control/Comparison	Duration of Intervention	Outcomes
Rifai et al., 2015 [25]	RCT	Patients with chronic HF (48)	Intervention: 60 ± 11 Comparison: 64 ± 12	DASH	General HF dietary recommendations	3 months	**Functional capacity** **6-min walk test (m):** Baseline: DASH = 254 ± 119 Comparison = 202 ± 77 *p* = 0.158 After 3 months: DASH = 292 ± 124 Comparison = 197 ±81 *p* = 0.018 **Quality of life (MLHFQ index)** Baseline: DASH = 29 ± 20 Comparison = 38 ± 4 *p* = 0.056 After 3 months: DASH = 21 ± 15 Comparison = 39 ± 22 *p* = 0.006
Silver, 2014 * [17]	RCT	Patients with chronic HF (40)	40 to 84	DASH	UD	30 days	**Functional capacity** **6-min walk test (m):** Baseline: DASH = 255 After 30 days: DASH = 292 *p* < 0.05 **Cardiac function (∆ units):** After 30 days: DASH = > 4 *p* < 0.05
Vittos et al., 2013 * [21]	Cohort	Hypertensive patients with compensated HFpEF (17) + healthy controls (10)	NR	DASH	DASH	21 days	**Oxidative stress** **MPO activity (UI/L):** Baseline: Intervention: 303.04 ± 18.7 Control: 248.0 ± 21.3 After 21 days: Intervention: 282.1 ± 14.9 *p* < 0.05 **BP (mmHg):** **Intervention group** Systolic Baseline: 158 After 21 days: 140 *p* <0.05 Diastolic Baseline: 81 After 21 days: 75 *p* < 0.05
Hummel et al., 2012 [23]	Cohort	Hypertensive patients with compensated HFpEF (13)	72 ± 10	DASH	NA	21 days	**Functional capacity** **6-min walk test (m):** Baseline: 313 ± 86 After 21 days: 337 ± 91 *p* = 0.006 **BP (mmHg):** Clinic Systolic: Baseline: 155 ± 29 After 21 days: 138± 22 *p* = 0.02 Clinic Diastolic: Baseline: 79 ± 15 After 21 days: 72 ± 8 *p* = 0.04 24-h ambulatory systolic: Baseline: 130 ± 4 After 21 days: 123 ± 4 *p* = 0.02 24-h ambulatory diastolic: Baseline: 67 ± 3 After 21 days: 62 ± 3 *p* = 0.02 **Oxidative stress** **Urinary F2-isoprostanes (pmol/mmol de Cr):** Baseline: 209 After 21 days: 144 *p* = 0.02
Hummel et al., 2013 [24]	Cohort	Hypertensive patients with compensated HFpEF (13)	72 ± 10	DASH	NA	21 days	**Cardiac function** **Arterial elastance (mmHg/mL):** Baseline: 2.0 ± 0.4 After 21 days: 1.7 ± 0.4 *p* = 0.007 **Ventricular-atrial coupling**: Baseline: 1.5 ± 0.3 After 21 days: 1.7 ± 0.4 *p* = 0.04 **Viscoelastic/relaxation flexibility (beats/min)**: Baseline: 24.3 ± 5.3 After 21 days: 22.7 ± 8.1 *p* = 0.03 **Chamber stiffness (s^-1^):** Baseline: 252 ± 115 After 21 days: 170 ± 37 *p* = 0.03
Hummel et al., 2014 * [20]	Cohort	Hypertensive patients with compensated HFpEF (12)	NR	DASH	NA	21 days	**Oxidative stress** F2-isoprostanes:Cr (pg/Mg): Baseline: 6.0 ± 2.5 After 21 days: 4.3 ± 1.4 *p* = 0.04 Aldosterone:Cr (pg/mg) Baseline: 6.7 ± 2.6 After 21 days: 14.2 ± 7.3 *p* = 0.009 Na:Cr (mg Na/mg Cr) Baseline: 59 ± 79 After 21 days: 30 ± 20 *p* = 0.01 ∆MBG:Cr × ∆Na:Cr β = 23.8 *p* < 0.001 ∆MBG:Cr × ∆F2-isoprostanes:Cr β = 1.1 *p* = 0.06
Chrysohoou et al., 2009a * [18]	Cross-sectional study	Patients with chronic HF (218)	M: 63 ± 13 W: 65 ± 13	Mediterranean diet	NA	NA	**Cardiac function** **Wave speed:** Smv = 0.62 ± 0.08, *p* = 0.001 Stv = 0.25 ± 0.09, *p* = 0.07 LVEF = 0.58 ± 0.30, *p* = 0.05 LAEF = 2.20 ± 0.67, *p* = 0.001 Emv = 0.69 ± 0.17, *p* = 0.001 Amv = 0.42 ± 0.20, *p* = 0.03
Chrysohoou et al., 2009b * [19]	Cross-sectional study	Patients with chronic HF (106)	65 ± 14	Mediterranean diet	NA	NA	**Inflammatory cytokines:** IL-6 = r = −0.56 ± 0.168 *p* = 0.004 TNF-a = r = −0.599 ± 0.281 *p* = 0.047 **Cytokines x Quality of life:** R = −0.52 ± 0.25, *p* = 0.040
Chrysohoou et al., 2012 [26]	Cross-sectional study	Patients with systolic HF (372)	M: 64 ± 13 W: 63 ± 13	Mediterranean diet	NA	NA	**Cardiac function** Before the adjusted analysis: log Smv: R = 0.154, *p* = 0.009 LAEF: R = 0.133, *p* = 0.041 Log E/AR = −0.24, *p* = 0.001 Log Emv/Amv: R = −0.133, *p* = 0.041 E/AxMDS: b =−0.225 ± 0.092, β = −0.782, *p* = 0.04
Levitan et al., 2013 [22]	Cohort	Postmenopausal women with HF (3.215)	50 to 79	Mediterranean and DASH diets	NA	4.6 years	**Mortality** **Mortality rate per 100 person-years 1:** Mediterranean diet Q1 (lower adherence): 10.1 Q4 (higher adherence): 8.0 HR 0.85 (IC 95% 0.70–1.02) *p* = 0.08 DASH diet Q1: 10.0 Q4: 8.8 HR 0.84 (IC 95% 0.70–1.00) *p* = 0.01
Gonzales et al., 2015 * [16]	RCT	Patients with stable chronic HF (123)	NR	Low-carb diet + exercise (group A) (*n* = 18):40% CHO, 40% LIP, 20% PTN Low-carb diet (group B) (*n* = 45):40% CHO, 40% LIP, 20%PTN	UD+ exercise (group C) (*n* = 21): 50% CHO, 30% LIP, 20%PTN UD (group D) (*n* = 39): 50% CHO, 30% LIP, 20% PTN	2 months	**Functional capacity** **6-min walk test (m):** Group A: Baseline: 270.37 ± 11.39 After 2 months: 301.78 ± 7.02 *p* = 0.08 Group B: Baseline: 345.77 ± 22.32 After 2 months: 370.07 ± 26.15 *p* = 0.05 **BP diastolic:** Group A: Baseline: 71.5 ± 2.98 After 2 months: 62.5 ± 1.70 *p* = 0.03 Group C: Baseline: 71.76 ± 2.31 After 2 months: 64.69 ± 1.9 *p* = 0.04
Evangelista et al., 2009 [27]	RCT	Ambulatory patients with HF, DM2 (untreated with insulin), overweight (BMI ≥ 27) and not eligible for transplantation (14)	58.8 ± 9.7	Hyperproteic and hypocaloric diet (HP) (*n* = 5): 40% CHO, 30% PTN, 30% LIP Normoproteic and hypocaloric (NP) (*n* = 5): 55% CHO, 15% PTN, 30% LIP	UD (*n* = 4)	12 weeks	**Functional capacity** **6-min walk test (m):** HP: 87.5± 21.0 NP: −3.7± 21.0 UD: −42.2 ± 23.5 *p* = 0.01 **VO_2_ max máximo (mL/kg/min):** HP: 3.1 ± 1.0 NP: −0.3 ± 1.0 UD: −0.3 ± 1.1 *p* = 0.003 **Quality of life (MLHFQ index):** MLHFQ general HP: −20.1 ± 9.5 NP: −12.2 ± 4.3 UD: −5.1 ± 3.9 *p* = 0.046

* Abstract; NA: not applicable; SD: study design; M: men; W: women; CHO: carbohydrate; DASH: dietary approaches to stop hypertension; DM: diabetes mellitus; EF: Ejection fraction; HF: Heart failure; HFpEF: Heart failure with preserved ejection fraction; HR: hazard ratio; LIP: lipids; LV: left ventricle; LA: left atrium; LVEF: ejection fraction of the left ventricle; LAEF: ejection fraction of left atrium; MLHFQ: Minnesota Living with Heart Failure Questionnaire; NR: not reported; PTN: protein; RCT: randomized clinical trial; BMI: Body mass index; UD: usual diet; HP: Hyperproteica and hypocaloric diet; NP: Normoproteic and hypocaloric.
